# Primary Mediastinal Germ Cell Tumor With Bone Marrow Infiltration: A Case Report and Literature Review

**DOI:** 10.1002/cnr2.70282

**Published:** 2025-07-19

**Authors:** Patricia Rioja, Guillermo Valencia, Zaida Morante, Renier Cruz, Tatiana Vidaurre, Silvia Neciosup

**Affiliations:** ^1^ Department of Medical Oncology Instituto Nacional de Enfermedades Neoplásicas (INEN) Lima Peru; ^2^ Oncosalud–AUNA Lima Peru; ^3^ Department of Pathology Instituto Nacional de Enfermedades Neoplásicas (INEN) Lima Peru

**Keywords:** bone marrow metastases, chemotherapy, extragonadal germ cell tumor, primary mediastinal germ cell tumor

## Abstract

**Introduction:**

Bone marrow infiltration is a rare presentation in patients with extragonadal germinal cell tumors (EGGCTs). We report a case of primary mediastinal germ cell tumor presenting with bone marrow metastases.

**Case Presentation:**

A 28‐year‐old male patient was admitted to Instituto Nacional de Enfermedades Neoplásicas with superior vena cava syndrome and a mediastinal mass accompanied by positive blood tumor markers for germinal cell tumor (GCT). In addition, he exhibited anemia and thrombocytopenia; therefore, bone marrow aspiration and biopsy were done and showed metastatic GCT cells. The patient started standard chemotherapy (bleomycin‐etoposide‐cisplatin) for 2 cycles; there was a reduction of serum tumor markers; however, the patient subsequently developed febrile neutropenia and severe pneumonia, resulting in death.

**Conclusion:**

This case demonstrates an uncommon presentation of EGGCT with bone marrow metastases. Early detection of this presentation will help to reduce the tumor burden and the mortality associated with it.

**Abbreviations**
AFP: alpha‐fetoprotein
autoHSCT: autologous stem cell transplant
BEP: bleomycin, etoposide, and cisplatin
CT: computerized tomography
DHL: lactic dehydrogenase
EGGCTs: extragonadal germinal cell tumors
EP: etoposide and cisplatin
GCT: germinal cell tumor
NS: nonseminomatous
OS: overall survival
PMCGT: primary mediastinal germ cell tumor
VIP: etoposide, ifosfamide, and cisplatin

## 1 Introduction

According to GLOBOCAN 2022, germ cell tumors have an estimated annual incidence of approximately 75 000 new cases and 9000 deaths [1]. GCTs represent 1% of all tumors and 5% of urological tumors. The presentation is common in males between 20 and 34 years old [2]. They are classified as extragonadal if there is no evidence of a primary tumor in the gonads (testes or ovaries). EGGCTs are an uncommon and heterogeneous group of neoplasms, comprising approximately 5%–10% of all GCTs. They tend to be more aggressive and have a poorer prognosis than gonadal GCTs, with an estimated overall survival (OS) of 25%. EGGCTs are mainly located in the midline of the body, from the brain to the coccyx [3]. Around 50% of patients with nonseminomatous (NS) primary mediastinal GCT (PMGCT) will present with metastatic disease at initial diagnosis.

EGGCTs spread via the lymphatic system to the anterior mediastinum (50%–70%), retroperitoneal lymph nodes (30%–40%), pineal gland (5%), and sacrococcygeal area (< 5%) [4]. Hematogenous spread is less common but has been reported to the lungs, brain, bone, and liver. Notably, bone marrow metastases are extremely rare, with only a few cases documented in the literature [5].

We report a case of a male patient with PMGCT, who was diagnosed with bone marrow infiltration.

## 2 Case Presentation

**FIGURE 1:**
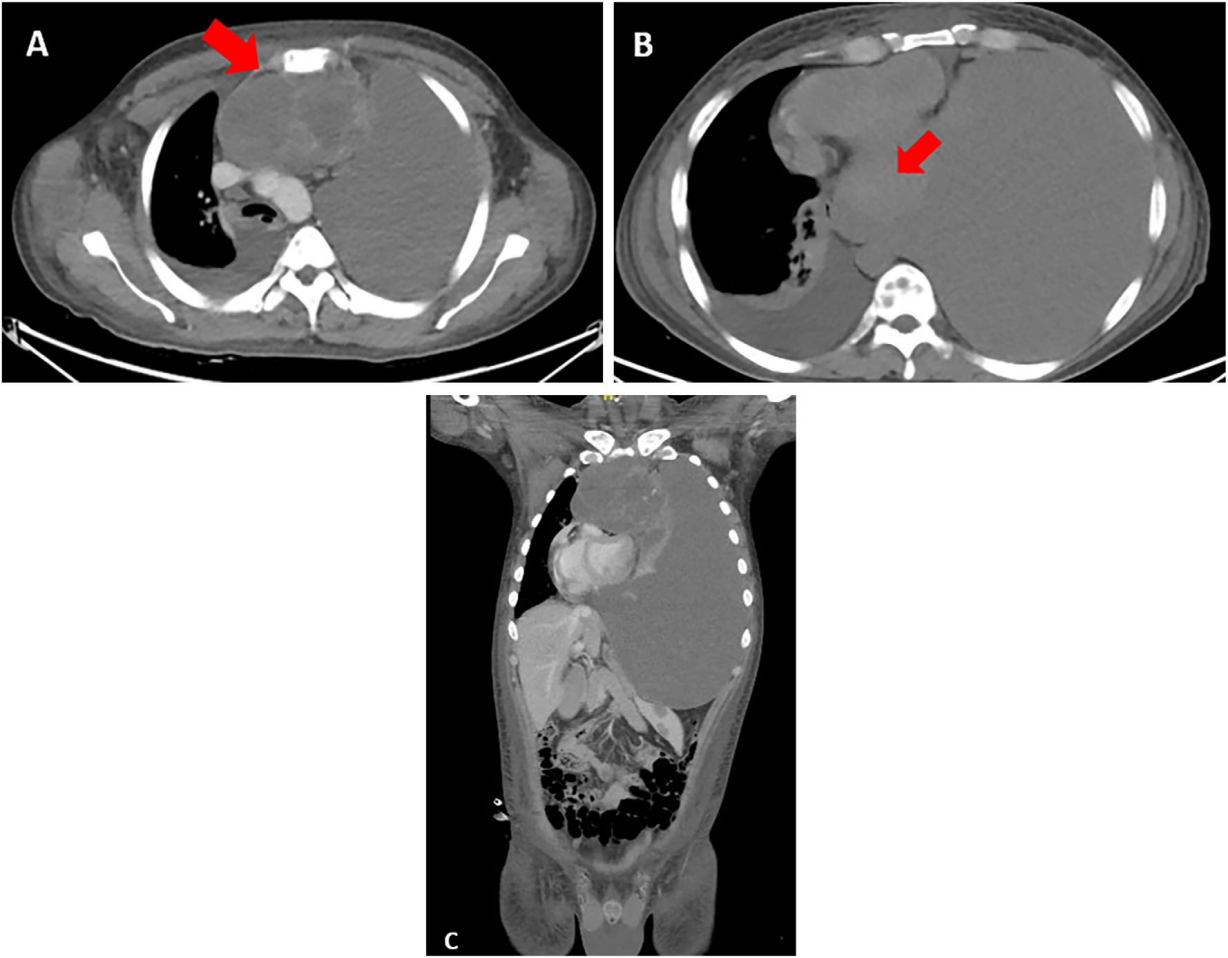
(A) Tumor with soft tissue density in the anterior mediastinum, retrosternal, prevascular, measuring 12.9 × 9.4 cm. (B) Left paracardiac mass measuring 70 × 55 mm. (C) Massive pleural effusion occupying the entire left hemithorax. Multiple bone metastases in the vertebral bodies.

**FIGURE 2:**
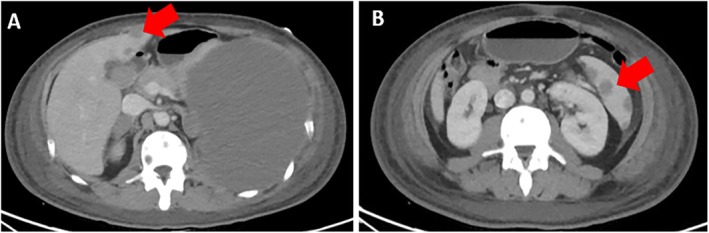
(A) Bone infiltration and multiple hepatic metastases (the largest measuring 12 mm). (B) Multiple splenic metastases (the largest measuring 20 mm).

**FIGURE 3:**
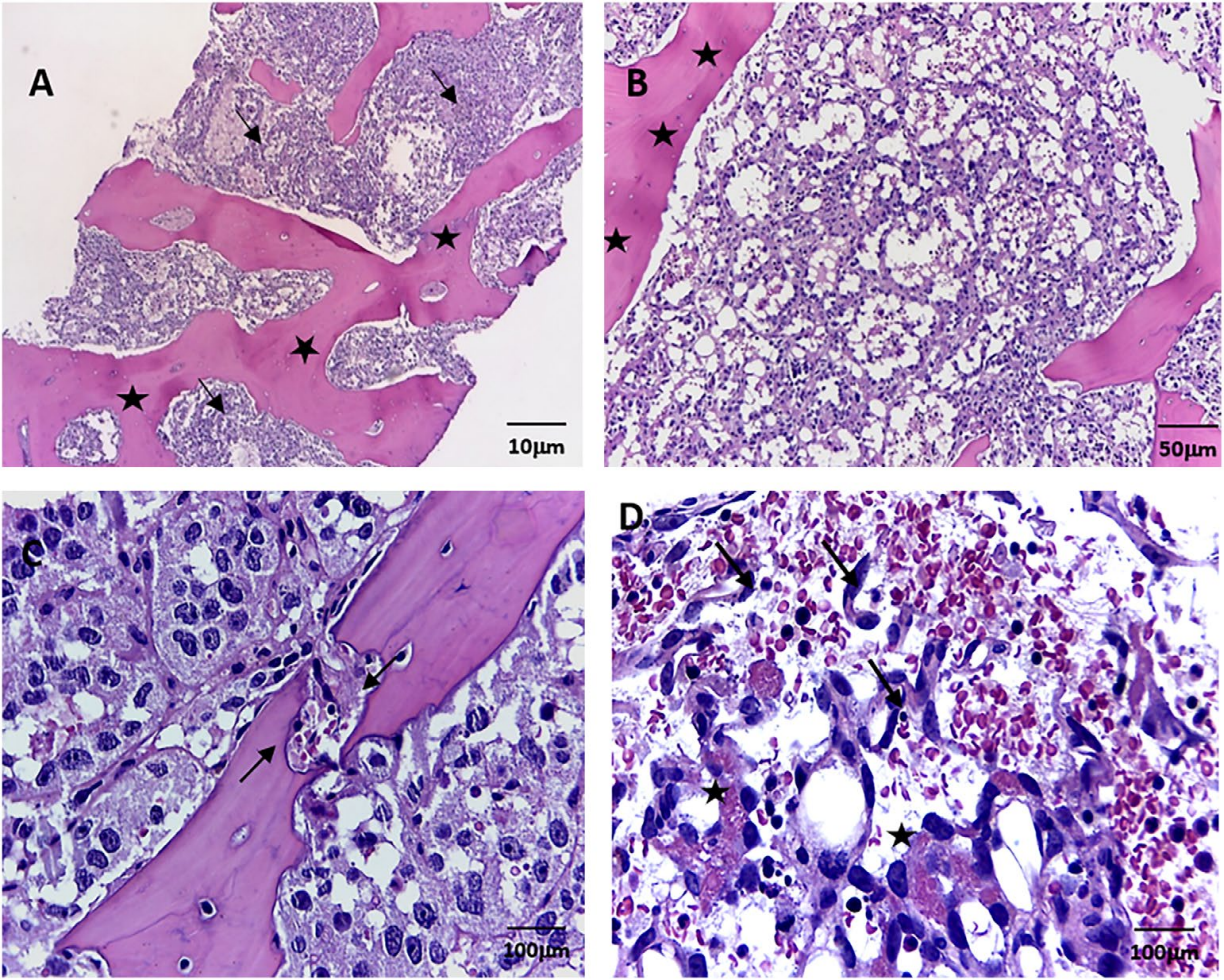
Bone marrow aspirate. Characteristics compatible with metastasis of germ cell tumor, yolk sac tumor type, microcystic variety. (A) Hematoxylin and eosin staining of bone marrow; ×10 magnification. (B) Hematoxylin and eosin staining of bone marrow; ×10 magnification. (C and D) Hematoxylin and eosin staining of bone marrow. Immunohistochemistry of bone marrow biopsy: SALL4−/+ (weak), CD30 (−), PLAP (−), SNF (−), AFP not evaluable, ki67: 80%; x100 magnification.

In March 2022, at Instituto Nacional de Enfermedades Neoplásicas, Lima, Peru, a 28‐year‐old male patient with no significant past medical history presented with 5 months of dyspnea on moderate exertion, hacking cough, anemia, and fatigue. Physical examination revealed superior vena cava syndrome, tachycardia, and a decrease in breath sounds in the left hemithorax. Chest computerized tomography (CT) demonstrated an anterior mediastinal tumor of 12.9 × 9.4 cm displacing large vessels and multiple metastatic bone lesions in dorsal and lumbar vertebrae (Figure 1). Abdominal CT revealed multiple hepatic metastases, splenic and bone infiltration, as well as hypodense lesions in both adrenal glands (the largest being 39 mm) (Figure 2). The testicular ultrasound examination was normal. Laboratory parameters revealed anemia of 7.3 g/dL (reference range 13.5–17.5 g/dL), thrombocytopenia of 24 000/μL (reference range 150 000–475 000/μL), serum alpha‐fetoprotein (AFP) level was 570.4 ng/mL (reference range < 7 ng/mL), beta‐human chorionic gonadotropin level was 6.75 μL (reference range < 2 ng/mL), and lactic dehydrogenase (DHL) was 3876 μL (reference range 112–456 μL). Liquid pleural cytology was suggestive of a germ cell tumor. A biopsy of the mediastinal mass revealed necrotic tissue, with a fragment of cartilaginous tissue exhibiting cellular atypia. This was consistent with a teratoma component of a germ cell tumor. The bone marrow aspirate was hypocellular, with 3% of the elements corresponding to neoplastic cells of non‐hematological origin. Histopathology of the bone marrow biopsy showed yolk sac tumor infiltration (Figure 3). Immuno‐histochemistry of the bone biopsy infiltrated by epithelioid, microcystic pattern neoplasia, positive for SALL4 and negative for CD30, placental alkaline phosphatase, and synaptophysin. Hence, the diagnosis was NS PMGCT metastatic to liver, left adrenal, bone, and bone marrow. In April 2022, the patient received the first cycle of standard chemotherapy, which consisted of bleomycin, etoposide, and cisplatin (BEP). Tumor markers normalization occurred after the first 2 cycles of chemotherapy (serum AFP level was 120 ng/mL, beta‐human chorionic gonadotropin level was 1.05 μL, and LDH was 470 μL). In July 2022, despite responding to initial chemotherapy, the patient developed respiratory symptoms, which worsened rapidly. He was admitted to the hospital for febrile neutropenia due to pneumonia and was subsequently transferred to the intensive care unit. He required mechanical ventilation but died of septic shock 2 weeks later.

## 3 Discussion

EGGCTs are uncommon neoplasms whose pathogenesis is poorly understood [6]. Bone marrow metastases are rare, and symptoms are nonspecific, with generalized bone pain and hematological abnormalities (leucopenia, anemia, and thrombocytopenia).

Klaassen et al. reported on three patients with NS PMGCT and bone marrow metastases. Two of the patients presented with severe thrombocytopenia, which is similar to our case report [7]. Zhou et al. analyzed the clinical outcomes of 30 patients diagnosed with bone marrow metastases stemming from solid tumors, noting that these cases were most prevalent in breast, lung, and gastrointestinal cancers. More than half of the patients presented with thrombocytopenia at the time of diagnosis, which correlated with a negative prognosis. The average survival time was merely 3 months, underscoring the grim outlook for individuals with bone marrow involvement [8].

Anner et al. examined 3620 bone marrow aspirates from patients diagnosed with solid tumors, including Ewing's sarcoma, squamous cell carcinoma of the lung, rhabdomyosarcoma, melanoma, prostate, and breast cancer. This study detected bone marrow infiltration in 20%–35% of these samples [9]. Although the incidence of bone marrow metastases may be elevated by the increased number of bone marrow smears, it is rarely positive for malignancy in biopsy. Overall, the presence of bone marrow metastases in solid tumors signifies a particularly unfavorable prognosis.

The first reported case of bone marrow metastasis in NS GCT occurred in 1854; however, the pathophysiology of this phenomenon remains uncertain [10]. Diagnosis is primarily based on elevated serum tumor markers and the suspicions of bone marrow infiltration in NS GCT cases.

Our case is of particular interest as NS GCT has been reported to cause bone marrow metastases in a limited number of cases based on available literature (Table 1) [10, 11]. In our patient, the bone marrow biopsy and immunochemistry were positive, even though case reports usually describe only suspected neoplastic cells in the bone marrow smear.

**TABLE 1:** Clinical features and outcomes in non‐seminomatous germ cell tumors with bone marrow infiltration.

	*N*	Years old	Histology	Site of primary	Site of metastases	Treatment	Outcome
Rioja et al. 2024	1	28	Non‐seminomatous germ cell tumor	Mediastinal	Bone marrow, liver, left adrenal and bone	BEP	Tumor marks response, died by infection.
Dubey, et al. 2023 [11]	1	NR	Non‐seminomatous germ cell tumor	Extragonadal	Lymph nodes, liver and bone marrow	BEP	In follow‐up
Ambreen, et al. 2021 [12]	1	14	Non‐seminomatous germ cell tumor	Mediastinal	Bone marrow	JEB and TIP	After 4 cycles no response.
Kumar, et al. 2016 [13]	1	21	Mixed germ cell tumor	Testis	Bone marrow	BEP	In follow‐up after 20 months of finish treatment
Garbay, et al. 2012 [10]	1	21	Non‐seminomatous germ cell tumor	Mediastinal	Bone marrow	BEP, TIP	Recurrence after 4 months to finish TIP
Orduz, et al. 2005 [14]	1	17	Non‐seminomatous germ cell tumor	Mediastinal	Bone marrow	High‐dose chemotherapy	No clinical response, died
Klaassen, et al. 1992 [7]	3	24, 33 and 48	Non‐seminomatous germ cell tumor	1st y 2nd patient: testis, 3th patient: extragonadal	Bone marrow	BEP	Relapse after treatment

Abbreviations: BEP, bleomycin, etoposide, and cisplatin; JEB, carboplatin, etoposide and bleomycin; *N*, number of patients; NR, not research; TIP, paclitaxel, ifosfamide and cisplatin.

Hematologic disorders (such as myelodysplasia or acute megakaryoblastic leukemia) are infrequently linked to PMNSGCT, though they have been consistently documented since 1985 [15] In such patients, the median time between the diagnosis of PMGCT and secondary blood malignancy is 5–6 months, with no patients surviving for more than 2 years. An abnormal isochromosome 12p may be found in leukemic blasts [16]. Our patient did not have a hematological malignancy detected on bone marrow biopsy, and cytogenetic analysis was not done. Second hematologic malignancies should be considered in the differential diagnosis of patients with PMGCT with cytopenia and bone marrow metastases [5].

There is a molecular association with bone marrow metastasis in PMGCTs. The CXCL12‐ XCR4 signaling pathway plays a role in inducing cells of different solid tumor types to metastasize to bone marrow [17]. Patients with PMGCTs have been shown to express CXCR4, the receptor for the chemokine CXCL12, while the mediastinum expresses CXCL12 during embryonic development [18].

All EGCT patients are treated as high‐risk GCTs, and 4 cycles of platinum‐based chemotherapy are recommended. Chemotherapy regimens include etoposide, ifosfamide, and cisplatin (VIP) for 4 cycles or BEP for 4 cycles, followed by surgical resection. The use of bleomycin is usually deferred to avoid lung toxicity. The use of high‐dose chemotherapy and autologous stem cell transplant (autoHSCT) is not recommended as it has not been evaluated in any prospective trial [19]. EGGCTs have a lower response rate to standard treatment than GCTs. PMGCTs have a poor prognosis, with a 40% OS in patients treated with VIP × 4, followed by surgical resection.

Surgical resection of residual disease after chemotherapy is mandatory in PMGCT. After a complete resection, 2 cycles of etoposide with cisplatin (EP) for 2 cycles are recommended for patients with residual viable GCT, while active surveillance is preferred in patients with only necrosis or teratoma in surgical tissue [20]. If surgery is not possible, a clinical trial or high‐dose chemotherapy followed by autoHSCT (with limited outcomes, 2‐year progression free survival of 23% vs. 63% in men with testicular GCTs) as second or third‐line options is recommended [21].

GCT patients with bone metastases have dismal outcomes (2‐year progression free survival and OS of 34% and 45%, respectively); a primary mediastinal tumor and the presence of liver and/or brain metastases were associated with a greater risk of progression or death [22]. The prognosis of PMGCT with bone marrow metastases is unfavorable in comparison with patients without infiltration. However, few case reports indicated that patients may respond to first‐line chemotherapy, with reduction of tumor markers to the normal range and clearance of tumor cells in bone marrow [10, 13]. Our patient received only BEP × 2, complicated by febrile neutropenia due to severe pneumonia, and died without completing the entire first‐line regimen.

This case highlights a rare presentation of EGGCT and the associated challenges with the clinical presentation. Additionally, this case supports the utility of bone marrow examination in the case of nonresponsive anemia and thrombocytopenia in a patient with PMGCT. However, this case report has several limitations: (1) The follow‐up duration is relatively short, only 5 months as of March 2022, limiting the assessment of long‐term disease‐free survival and OS; (2) the findings of this study may not be generalizable. (3) Potential biases involved researchers may influence the conclusions of the study.

## 4 Conclusion

PMGCT with bone marrow metastases is extremely rare. Pathophysiology involves molecular alterations that induce migration of cells to the bone marrow. It is mandatory to explore the bone marrow (aspiration and biopsy) in a PMGCT with cytopenia, as secondary blood malignancies may also occur. EGGCTs are treated as high‐risk NS GCT (4 cycles of platinum‐based regimens), and outcomes are poorer with standard chemotherapy compared with testicular GCTs.

## Author Contributions

All authors had full access to the data in the study and take responsibility for the integrity of the data and the accuracy of the data analysis. Conceptualization: Patricia Rioja. Methodology: Renier Cruz, Guillermo Valencia, and Zaida Morante. Resources: Tatiana Vidaurre. Writing: Original Draft: Patricia Rioja. Writing: Review and Editing: Patricia Rioja, Guillermo Valencia, and Renier Cruz. Visualization: Tatiana Vidaurre; Supervision: Silvia Neciosup.

## Ethics Statement

This report adheres to the journal's patient consent policy, with the patient's written authorization for publication secured.

## Consent

A written informed consent was obtained from the patient for publication of this case report and any accompanying images. A copy of the written consent is available for review.

## Conflicts of Interest

The authors declare no conflicts of interest.

## Data Availability

Data sharing is not applicable to this article as no new data were created or analyzed in this study.

## References

[1] F. Bray , M. Laversanne , H. Sung , et al., “Global Cancer Statistics 2022: GLOBOCAN Estimates of Incidence and Mortality Worldwide for 36 Cancers in 185 Countries,” CA: A Cancer Journal for Clinicians 74, no. 3 (2024): 229–263. 38572751 10.3322/caac.21834

[2] “Testicular Cancer–Introduction–Uroweb,” https://uroweb.org/guidelines/testicular‐cancer.

[3] J. Oldenburg , D. M. Berney , C. Bokemeyer , et al., “Testicular Seminoma and Non‐Seminoma: ESMO‐EURACAN Clinical Practice Guideline for Diagnosis, Treatment and Follow‐Up,” Annals of Oncology 33, no. 4 (2022): 362–375. 35065204 10.1016/j.annonc.2022.01.002

[4] “Extragonadal Germ Cell Tumors Clinical Presentation: History, Physical Examination, Complications,” https://emedicine.medscape.com/article/278174‐clinical?utm_source=chatgpt.com.

[5] T. Ueda , I. Takada , T. Shimizu , et al., “Bone Marrow Metastasis in a Patient With Non‐Seminomatous Testicular Germ Cell Tumor,” IJU Case Reports 5 (2022): 247–250. 35800110 10.1002/iju5.12446 PMC9249660

[6] L. Iorga , D. Marcu , O. Bratu , et al., Extragonadal Germ‐Cell Tumors–A Review of the Pathogenesis, Histopathological Findings, Diagnosis and Treatment, vol. 54 (Archives of the Balkan Medical Union. Balkan Medical Union, 2019), 725–730.

[7] U. Klaassen , C. Bokemeyer , and H. J. Illiger , “Metastatic Bone Marrow Involvement in Malignant Germ Cell Tumors,” Oncology Research and Treatment 15, no. 5 (1992): 394–396, https://www.scienceopen.com/document?vid=5daae396‐abfb‐44c7‐9d4f‐cf9a036ef553.

[8] M. H. Zhou , Z. H. Wang , H. W. Zhou , M. Liu , Y. J. Gu , and J. Z. Sun , “Clinical Outcome of 30 Patients With Bone Marrow Metastases,” Journal of Cancer Research and Therapeutics 14 (2018): S512–S515. 29970716 10.4103/0973-1482.172717

[9] R. M. Anner and B. Drewinko , “Frequency and Significance of Bone Marrow Involvement by Metastatic Solid Tumors,” Cancer 39 (1977): 1337–1344. 912663 10.1002/1097-0142(197703)39:3<1337::aid-cncr2820390349>3.0.co;2-x

[10] D. Garbay , F. Durrieu , B. Bui , and A. Italiano , “Bone Marrow Metastases in a Patient With Primary Mediastinal Non‐Seminomatous Germ Cell Tumor—An Unusual Pattern of Relapse,” Oncology Research and Treatment 35, no. 1–2 (2012): 40–42, https://www.karger.com/Article/FullText/335881. 10.1159/000335881 22310344

[11] H. Dubey , G. Jain , C. Kumar , et al., “Bone Marrow Metastasis of Testicular Germ Cell Tumour: A Rare Case,” Heliyon 9, no. 6 (2023): e16703. 37303538 10.1016/j.heliyon.2023.e16703 PMC10248114

[12] F. Ambreen , S. Shaikh , F. Meraj , M. Mushtaq , and M. Ashraf , “Relapsed Malignant Germ Cell Tumor Metastasized in Bone Marrow: a Rare Find‐ing,” JSM Clinical Case Reports 9, no. 2 (2021): 1190.

[13] U. Kumar , P. Ramteke , P. Das , A. Gogia , and P. Tanwar , “Isolated Bone Marrow Metastasis of Testicular Tumor: A Rare Cause of Thrombocytopenia,” Urology Annals 9, no. 1 (2017): 96. 28216942 10.4103/0974-7796.198833 PMC5308052

[14] R. Orduz , E. Sabattini , F. Bacci , et al., “Pitfalls in Diagnosis: Primary Mediastinal Non‐Seminomatous Germ Cell Tumour With Bone Marrow Metastasis Showing Melanoma‐Like Phenotype [3],” Histopathology 47, no. 6 (2005): 645–646. 16324208 10.1111/j.1365-2559.2005.02167.x

[15] H. Yaegashi , T. Nohara , K. Shigehara , et al., “Survival Outcomes of Patients With Primary Mediastinal Germ Cell Tumors: A Retrospective Single‐Institutional Experience,” Cancer Diagnosis and Prognosis 2 (2022): 352–359. 35530648 10.21873/cdp.10116 PMC9066546

[16] R. K. Bharat vignesh , M. S. Latha , S. Sri Gayatri , J. Dhaarani , and F. Andrea Mary , “Acute Myeloid Leukemia Following a Primary Mediastinal Germ Cell Tumor in an Adolescent Boy: A Case Report,” Pediatric Hematology Oncology Journal 8 (2023): 191–193.

[17] G. Smaldone , F. Di Matteo , R. Castelluccio , et al., “Targeting the CXCR4/CXCL12 Axis in Cancer Therapy: Analysis of Recent Advances in the Development of Potential Anticancer Agents,” Molecules 30 (2025): 1380. 40142155 10.3390/molecules30061380 PMC11945090

[18] Y. Shi , D. J. Riese , and J. Shen , “The Role of the CXCL12/CXCR4/CXCR7 Chemokine Axis in Cancer,” Frontiers in Pharmacology 11 (2020): 574667. 33363463 10.3389/fphar.2020.574667 PMC7753359

[19] J. Chahoud and M. Kohli , “Managing Extragonadal Germ Cell Tumors in Male Adults,” AME Medical Journal 5 (2020): 8.

[20] T. D. Gilligan , “Resection of Residual Masses After Chemotherapy for Metastatic Nonseminomatous Germ Cell Tumors in Adolescents and Adults,” Journal of Clinical Oncology 41 (2023): 3899–3904. 37410968 10.1200/JCO.23.00654

[21] J. Briones , P. Diaz , and B. D. Nicholson , “High‐Dose Chemotherapy as Initial Salvage Chemotherapy in Patients With Relapsed or Refractory Testicular Cancer: A Systematic Review and Meta‐Analysis,” Frontiers in Oncology 14 (2024): 1437574. 39411122 10.3389/fonc.2024.1437574 PMC11473300

[22] R. Gille , B. Allignet , F. Izarn , P. Peyrat , H. Boyle , and A. Fléchon , “Bone Metastases in Non‐Seminomatous Germ Cell Tumors: A 20‐Year Retrospective Analysis,” Journal of Clinical Medicine 13 (2024): 3280. 38892991 10.3390/jcm13113280 PMC11172778

